# Tablet-Based Strength-Balance Training to Motivate and Improve Adherence to Exercise in Independently Living Older People: Part 2 of a Phase II Preclinical Exploratory Trial

**DOI:** 10.2196/jmir.3055

**Published:** 2014-06-25

**Authors:** Eva van het Reve, Patrícia Silveira, Florian Daniel, Fabio Casati, Eling D de Bruin

**Affiliations:** ^1^Institute of Human Movement Sciences and SportDepartment of Health Sciences and TechnologyETH ZurichZurichSwitzerland; ^2^University of TrentoDepartment of Information Engineering and Computer ScienceUniversity of TrentoTrentoItaly; ^3^CAPHRI School for Public Health and Primary CareDepartment of EpidemiologyMaastricht UniversityMaastrichtNetherlands; ^4^Centre for Evidence Based PhysiotherapyMaastricht UniversityMaastrichtNetherlands

**Keywords:** gait, aging, exercise therapy, tablet, delivery of health care

## Abstract

**Background:**

Home-based exercise programs can improve physical functioning and health status of elderly people. Successful implementation of exercise interventions for older people presents major challenges and supporting elderly people properly while doing their home-based exercises is essential for training success. We developed a tablet-based system—ActiveLifestyle—that offers older adults a home-based strength-balance training program with incorporated motivation strategies and support features.

**Objective:**

The goal was to compare 3 different home-based training programs with respect to their effect on measures of gait quality and physical performance through planned comparisons between (1) tablet-based and brochure-based interventions, (2) individual and social motivation strategies, and (3) active and inactive participants.

**Methods:**

A total of 44 autonomous-living elderly people (mean 75, SD 6 years) were assigned to 3 training groups: social (tablet guided, n=14), individual (tablet guided, n=13), and brochure (brochure guided, n=17). All groups joined a 12-week progressive home-based strength-balance training program. Outcome measures were gait performance under single and dual task conditions, dual task costs of walking, falls efficacy, and physical performance as measured by the Short Physical Performance Battery (SPPB). Furthermore, active (≥75% program compliance) and inactive (<75% program compliance) individuals were compared based on their characteristics and outcome measures.

**Results:**

The tablet groups showed significant improvements in single and dual task walking, whereas there were no significant changes observable in the brochure group. Between-groups comparisons revealed significant differences for gait velocity (*U=*138.5; *P*=.03, *r=*.33) and cadence (*U=*138.5, *P*=.03 *r=*.34) during dual task walking at preferred speed in favor of the tablet groups. The brochure group had more inactive participants, but this did not reach statistical significance (*U=*167, *P*=.06, *r=*.29). The active participants outperformed the inactive participants in single and dual task walking, dual task costs of walking, and SPPB scores. Significant between-groups differences were seen between the tablet groups and the brochure group, in favor of the tablet groups.

**Conclusions:**

A tablet-based strength-balance training program that allows monitoring and assisting autonomous-living older adults while training at home was more effective in improving gait and physical performance when compared to a brochure-based program. Social or individual motivation strategies were equally effective. The most prominent differences were observed between active and inactive participants. These findings suggest that in older adults a tablet-based intervention enhances training compliance; hence, it is an effective way to improve gait.

## Introduction

One of the major opportunities to extend years of active independent life and to promote an independent lifestyle is to be physically active on a regular basis [1-3]. Physical activity can prevent several diseases (eg, cancer and type II diabetes mellitus) and has the potential to enhance both physical and cognitive functioning and cardiovascular and psychological health [4-8]. For older adults, structured exercise training is recommended to postpone frailty and vulnerability [9,10] and to minimize several chronic degenerative diseases that result from an inactive lifestyle [11,12]. The evidence that sedentary people lose a relatively large fraction of their muscle mass in the aging process [1] confirms that avoiding physical inactivity is an obvious aim for interventions [12]. In past research, the effect of physical activity has also been linked to an increase in life expectancy [13], diminishing probability of a fall [14-16], and in preventing sarcopenia [1,17]. Accordingly, keeping older adults physically active is a crucial step toward prevention of several diseases.

Walking requires efficient circulatory, heart, lung, nervous, and musculoskeletal systems [18]. In combination with deficits in these systems, walking ability in old age often deteriorates. Gait quality assessment with dedicated gait analysis systems [19], expressed through measures of variability [20], showed that both stride time and stride length variability are associated with the control of the rhythmic stepping mechanism [21]. Errors in foot placement control and/or displacement of the center of mass may result in higher variability [21,22], which in turn leads to falls in older adults [23,24]. Furthermore, gait speed is one of the factors reflecting functional ability and independence [25]. Slowed walking may reflect damaged organ systems [18,26]. Relationships between faster gait speed and reduced mortality [18,27], and shorter length of stay in geriatric hospitals [28,29] have been demonstrated. In contrast, reduced gait speed can be associated with falls and a decline in cognitive factors [30], such as attention and psychomotor speed [31]. This reduction in gait speed in older people has been shown to be a result of shorter step lengths [32,33] and increased double support time [33,34], which are changes in gait that relate to falls in elderly [35]. Another important determinant of gait function in both healthy and unhealthy elderly is lower extremity muscle function [36]. To summarize, a decrease in walking ability and abnormal walking frequently results in disability and falls, which can lead to a loss of independence in activities of daily living [37], institutionalization, and death [38,39]. Furthermore, a lack in gait quality can lead to a fear of falling [37].

When strength training is complemented with balance exercises, a transfer to functional tasks of daily living may be expected [40]. Therefore, to optimize walking quality, strength and balance training, previously showed to be effective [41-43], should be applied and adhered to.

Home-based exercise programs—provided that they are performed correctly—can be effective in improving physical functioning [15] and health status of elderly people [1]. Especially for older adults without access to exercise facilities (eg, because they live in rural or remote areas), an effective home-based intervention at regular intervals potentially offers great benefits. Large travel distances and deteriorations in locomotion could potentially limit the ability of these people to visit a training center [44,45]. However, despite the fact that exercise has been widely accepted as beneficial for health, successful implementation of exercise interventions presents a major challenge for many older people [46].

The importance of monitoring and supporting elderly people while doing their home-based exercises should not be neglected. Providing feedback, social support, motivation, and encouragement seem to be essential factors in enhancing adherence [47-49]. Although older adults often express the desire for training support at home [50], these factors are difficult to implement in home-based exercise programs. Remote feedback strategies may have the potential to replace live supervision while exercising at home [51]. For an overview about related work on this topic, we refer to our previous studies describing our phase II study [52] with the tablet-based app ActiveLifestyle [53]. This part of our study compares 3 different home-based training programs and their effect on measures of gait quality while considering adherence to the training program. We hypothesized differing results for (1) tablet-based groups when compared to a brochure group, (2) a tablet group with social motivation strategies when compared to a tablet group with individual motivation strategies, and (3) active participants when compared to inactive participants.

## Methods

### The ActiveLifestyle App

ActiveLifestyle offers autonomous-living older adults tablet-based software that supports them doing their physical exercises. The app assists, monitors, and motivates this group of people while doing their exercise program at home. The program consists of a strength and balance training plan. Exercises are shown with videos and explained with written and oral instructions. Details of the exercises are given in the Intervention Program section. The ActiveLifestyle app comes in 2 different versions: the individual version contains individual motivation strategies and the social version consists of individual and social motivation strategies. Social and individual motivation strategies were included to help participants comply with the training plan. A summary description of these motivation strategies is provided here because a detailed description has been published elsewhere [53]. The intention of the integrated individual motivation strategies (ie, conditioning through positive and negative reinforcement, goal setting, self-monitoring, awareness) is to convince the person about the expected gain for himself/herself (eg, enhance awareness of health benefits by doing strength and balance exercises). Social motivation strategies (ie, comparison, external monitoring, emotional support, collaboration) aim at supporting individuals (eg, through a social network consisting of training partners and caregivers). ActiveLifestyle supports 6 main features accessible through its menu:

1. The What’s Next? option invites the users to start the performance of due workout sessions.
2. The Weekly Exercises option shows the scheduled strength-balance sessions organized per week.
3. The Progress option shows the user’s progress through the conditioning, goal setting, and self-monitoring strategies previously mentioned in both versions. The social version, in addition to these strategies, also supports the collaboration strategy through a collaborative game.
4. The Bulletin Board allows the users to receive written messages, which may include links to websites and YouTube videos. Three types of messages are supported: (1) workout session completed messages to inform the participant(s) about the conclusion of a scheduled session of exercises, (2) ActiveLifestyle tips messages to support the awareness motivation strategy, and (3) public messages written by the training members. It is important to note that only the social version supports the third type of messages and can send messages to the whole training plan community.
5. The Friends option lists the members of the training plan community (ie, older users and experts). Only the social version supports this feature.
6. The inBox option allows users of the social version to exchange private text messages with their list of friends.

To minimize failure to follow the program because of a memory lapse, an alarm clock helps to remind participants about their training thrice daily. The application has previously shown to be feasible for older adults [54].

### Participants

A sample of 44 autonomously living older adults was selected according to the following inclusion criteria: older than 65 years, able to walk 20 meters with or without aids, and free of rapidly progressive illness, acute illness, or unstable chronic illness. Ethical approval for the study was obtained from the ETH Ethics Committee (EK 2011-N-64). All participants provided written consent before they participated in the study.

Participants were recruited by convenience sampling from 2 institutions for older people and 1 organization responsible for coordinating and providing at-home nursing care for seniors. The Senioren Begegnungszentrum Baumgärtlihof, a day center dedicated to deliver services and information related to the older population (Horgen, Switzerland), advised potential participants through its mailing list and by notes in the local newspaper. The Alterswohnungen Turm-Matt, a cooperative offering housing and daily living facilities to older people (Wollerau, Switzerland), informed and advised potential participants in person or by phone and distributed flyers to advertise the study. The Fachstelle für präventive Beratung Spitex-Zürich, a home-care nursing organization (Spitex-Zürich), promoted the study sending letters and specifically inviting patients in need of better physical performance. Spitex nurses selected potential participants based on the eligibility criteria.

Participants who fulfilled the inclusion criteria were assigned to either the brochure group (n=17), the social group (n=13), or the individual group (n=14). The social and individual groups received a tablet with the ActiveLifestyle app. Both the social and individual group versions of the app consisted of individual motivation strategies, whereas social motivation strategies were added only for the social group. Participants in the brochure group did their exercises using a training plan on paper sheets.

Participants who stopped doing their exercises during the 12 weeks of the program were defined as dropouts.

### Design

#### Overview

**Figure 1:**
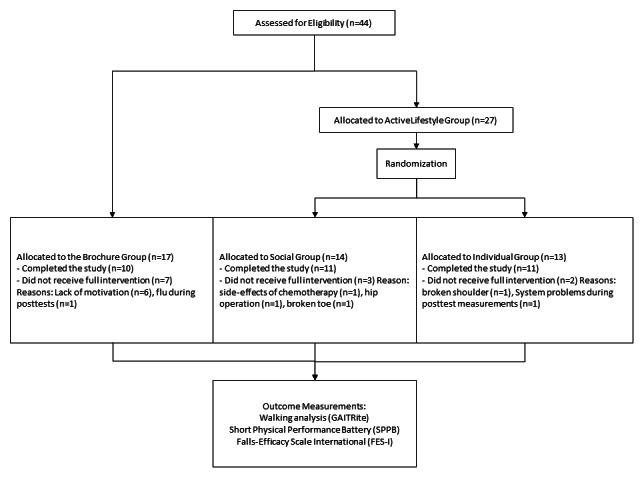
The study flowchart.

This study was designed as a phase II preclinical exploratory trial. The outcome variables were measured at baseline (T0) and after 3 months of the intervention. Pre and post measurements took place in suitable locations at the participating institutions. Individuals from the tablet groups visited 1 class to learn how to use the tablet and the included ActiveLifestyle app. A second class was held for all 3 groups to give instructions on how to do the exercises. The training exercise program was to be conducted at home. A flowchart of participants is presented in Figure 1.

At entry to the study, a medical history through self-report was taken for demographic and health-related information.

#### Motor Intervention Program

**Figure 2:**
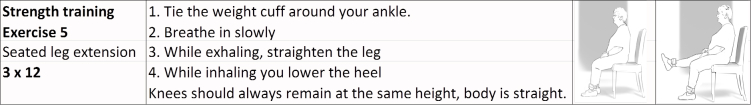
Example of an exercise instruction: intermediate seated leg extension with weights.

Interventions that aim to improve walking function and prevent falls should include both strengthening and balance exercises [55]. Therefore, we developed a program with the help of guidelines and recommendations from previous studies [12,56-58]. Participants from all groups performed the same strength and balance exercises. Detailed information about the physical exercises is shown in Table 1 and an exercise example is given in Figure 2.

The intervention consisted of twice-weekly progressive resistance training for 12 weeks. Training devices used were resistance bands and soft balls. Exercises with resistance bands showed to be efficient in enhancing physical functioning in autonomously living older adults [59]. Before the strength exercises, participants conducted a warm up. Flexibility exercises followed the program to maintain or improve range of motion necessary for daily activities. Additionally, all participants performed 3 balance exercises 5 times a week. Frequency, intensity, and duration of the exercises were based on published recommendations [12,56,58,60].

**Figure 3:**
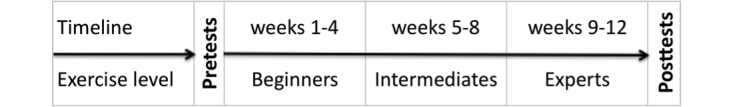
Timeline and exercise levels.

To ensure exercise progression during the whole program, the intervention was divided into 3 levels (Figure 3). From week 1 to week 4, participants trained at the beginner level; from week 5 to week 8, they trained at the intermediate level; and from week 9 to week 12, they trained at the expert level. The 3 training levels differed in exercise execution, number of sets, and training additives (eg, ankle weights for strength exercises, towels for balance exercises).

Following performance of each strength and balance exercise, participants registered their performed sets, repetitions, and perceived exertion on Borg’s scale of perceived exertion [61]. The social and individual groups were automatically asked to provide feedback of their exercise experience in the app. Without this feedback, the program would not continue. The brochure group received a paper form to provide this feedback information with a pencil. This information was expected following each strength and balance exercise.

**Table 1:** Exercises of the intervention program.

Exercises	Beginner	Intermediate	Expert
Warm up; 2 times/week	1 set, 10 repetitions	1 set, 10 repetitions	1 set, 10 repetitions
	Shoulder rotation	Standing shoulder rotation	Standing shoulder rotation
	Arms circles	Standing arms circles	Standing arms circles
	Leg raises	March	March
	Pointed foot to heel	Standing foot to heel point	Standing foot to heel point
	Hip abduction and adduction	Standing side tap	Standing side tap
	Rest head left and right	Rest head left and right	Rest head left and right
Strength training; 2 times/week	2 sets, 12 repetitions	3 sets, 12 repetitions	3 sets, 12 repetitions
	Chair stand	Chair stand, arms stretched out	Fast chair stand
	Seated hip flexion	Standing hip flexion	Standing hip flexion without placing foot on the floor
	Seated hip adduction	Seated hip adduction	Standing hip adduction
	Seated hip abduction	Standing hip abduction^a^	Standing hip abduction without placing foot on floor^a^
	Seated leg extension	Seated leg extension^a^	Standing leg extension^a^
	Standing leg curl	Standing leg curl a	Standing leg curl a, without placing foot on floor
	Standing heel lift	Standing heel lift^a^	One-leg heel lift^a^
	Seated sit-ups	Seated sit-ups, arms behind head	Seated sit-ups, straight arms overhead
	Seated side arm raise with resistance band	Standing side arm raise with resistance band	Side arm raise with resistance band, fast movement
	Seated toe lift	Seated toe lift^a^	Standing toe lift^a^
Stretching; 2 times/week	3 sets, 15 seconds	3 sets, 15 seconds	3 sets, 15 seconds
	Seated leg stretch	Seated leg stretch	Seated leg stretch
	Seated hip stretch	Seated hip stretch	Seated hip stretch
Balance; 5 times/week	3 sets, 15 seconds	3 sets, 15 seconds	3 sets, 15 seconds
	One-leg stand	One-leg stand on a towel	One-leg stand, eyes closed
	Full tandem stand	Full tandem stand on a towel	Full tandem stand, eyes closed
	Heel-to-toe walk	Heel-to-toe walk, forward and backward	Heel-to-toe walk, eyes closed

^a^With ankle weights (0.5-2 kg per leg).

### Test Procedures and Outcome Measures

#### Program Adherence

The criteria for success of our pilot study [62] were based on feasibility objectives and focused on compliance with the training plan (eg, the attendance rate of participants). For adherence to the intervention, the compliance of the participants with all trainings was recorded. A compliance rate of 75% was deemed acceptable [63]. Participants were defined as active participants when 75% or more of all planned exercises were performed or as inactive with an attendance less than 75% [64]. Compliance within the exercise program for the groups using tablets was assessed with an automatic registration after completing the exercises, whereas participants of the brochure group had to fill in a training plan diary.

#### Gait Analysis

Gait was measured with the portable electronic GAITRite walkway with Platinum Version 4.0 software (CIR Systems, Sparta, NJ, USA). Sampling rate was 60 Hz [65,66]. This system is a valid and reliable tool for measuring spatial and temporal parameters of gait [67]. Participants were instructed to walk under 4 conditions for 2 or 3 trials each, depending on their physical condition: (1) at their self-selected speed (preferred walking), (2) at their fastest speed (fast walking), (3) at their self-selected speed while concurrently performing a cognitive task (dual task preferred walking), and (4) at their fastest speed while concurrently performing a cognitive task (dual task fast walking). For the cognitive task, participants were asked to continuously subtract 7 or 3 from a given number while walking. If they were not able to perform the cognitive task, the arithmetical task was modified to a verbal fluency task. The verbal fluency task consisted of enumerating animals or flowers. Participants were asked not to give priority to one task over the other in the dual task test condition, but to try to perform both (walking and calculating) equally well at the same time.

The following temporal-spatial parameters were taken for analysis: velocity (cm/s), cadence (steps/min), step time (s), step length (cm), double support time (s), and variability of step time and length. Variability was expressed as standard deviation of step time (SD step time) and standard deviation of step length (SD step length) over the measured number of gait cycles while walking on the GAITRite walkway.

To quantify participants’ ability to execute 2 tasks simultaneously, we calculated the relative dual task costs (DTC) of walking according the following formula [68,69]: DTC (%)=100 * (single task score-dual task score) / single task score.

#### Physical Performance and Fear of Falling

Lower extremity functioning was assessed with the Short Physical Performance Battery (SPPB). This test battery consists of a balance test, a 3-meter gait test, and a 5 chair-rises test. The sum of the 3 components comprises the final SPPB score with a possible range from 0 to 12 (12 indicating the highest degree of lower extremity functioning). The SPPB is a valid and reliable measure of mobility in older adults [70] and can predict future disability [71].

The Falls Efficacy Scale International (FES-I) questionnaire was used as a measure of concern about falling to determine the transfer effects of training. This scale assesses both easy and difficult physical activities and social activities (scale: 1=not at all concerned, 2=somewhat concerned, 3=fairly concerned, 4=very concerned). The FES-I has excellent internal and test-retest reliability [72].

### Statistical Analysis

All statistical procedures were conducted with SPSS version 21.00 (SPSS Inc. Chicago, IL, USA). All available data were analyzed by initial group assignment and were performed with an intention-to-treat approach [73]. All participants were included in the analysis regardless of their adherence rate. We assumed that all missing responses were constant and replaced the missing values with mean values of the group to which participants were originally allocated [74]. Because of the nonnormality of the data, baseline comparison and interaction effects of groups (between-groups differences) were undertaken using a Mann-Whitney *U* test. The effects size, *r*, was calculated as *r=z*/√N (where *z* is the approximation of the observed difference in terms of the standard normal distribution and N is the total sample number). To identify differences between pretests and posttests (within-group differences) a Wilcoxon signed rank test was conducted. We identified differences between (1) the brochure group and the tablet groups (brochure group vs social and individual groups), (2) between the 2 tablet groups: (social group vs individual group), and (3) between active and inactive participants using planned comparisons.

Suggested norms for interpreting *r* are .10=small effect, .30=moderate effect, and .50=large effect. A probability level of *P*<.05 was considered to be statistically significant.

## Results

### Overview

Participants’ demographics and baseline characteristics are summarized in Table 2. Results are based on a self-report health questionnaire. In general, there were no significant between-groups differences of baseline demographics for most parameters using planned comparisons: (1) brochure group vs social group and individual groups and (2) social group vs individual group. We detected significant differences between (1) brochure group vs social and individual groups in joint diseases, practiced some sport in the past, and 3 or more medications daily. The brochure group reported less joint diseases (*U=*139, *P*=.01, *r=*.38), practiced less sport in the past (*U=*144, *P*=.02, *r=*.36), and took less medication (*U=*166.5, *P*=.04, *r=*.32).

**Table 2:** Participants’ demographics and baseline characteristics (N=44).

Characteristic			Group			Activity level	
			Brochure n=17	Social n=13	Individual n=14	Active n=26	Inactive n=18
Age, mean (SD)			76 (15)	74 (5)	75 (6)	75 (5)	76 (7)
**Sex, n (%)**							
	Female		10	8 (62)	10 (71)	15 (58)	13 (72)
	Male		7	5 (38)	4 (29)	11 (42)	5 (28)
**Fall risk factors, n (%)**							
	Slow walking speed (<1.22 m/s)		10 (59)	9 (69)	7 (50)	14 (54)	12 (67)
	Fell in the last 6 months^a^		4 (24)	5 (38)	2 (14)	5 (19)	6 (33)
	3 or more prescription medications		1 (6)	4 (31)	5 (36)	8 (30)	2 (11)
	Physical functioning; SPPB (points)		9.8	9.7	9.9	10.1	9.4
	Fear of falling; FES-I (points)		17.9	20.0	18.5	18.9	18.9
**Education/profession, n (%)**							
	University/college		1 (6)	2 (15)	3 (21)	3 (12)	3 (17)
	Vocational education		10 (59)	7 (54)	7 (50)	18 (69)	8 (44)
	No educated profession		6 (35)	4 (31)	4 (29)	5 (19)	7 (39)
	In a sitting position past profession		6 (35)	6 (46)	7 (50)	13 (50)	8 (44)
**Health questions, n (%)**							
	**Number of self-reported chronic diseases**						
		Joint diseases	4 (24)	7 (54)	9 (64)	13 (50)	8 (44)
		Hypertension	5 (29)	4 (31)	3 (21)	8 (31)	4 (22)
		Cardiac problems	3 (18)	4 (31)	4 (29)	6 (23)	5 (28)
		Osteoporosis	3 (18)	2 (15)	2 (14)	3 (12)	4 (22)
		Type II diabetes mellitus	1 (6)	1 (8)	3 (21)	1 (4)	5 (28)
	**Self-reported walking problems**		5 (29)	6 (46)	3 (21)	8 (31)	6 (33)
		Need walking aid	1 (6)	2 (15)	1 (7)	3 (12)	1 (6)
	Hearing problems		6 (35)	5 (38)	4 (29)	8 (31)	7 (39)
	Vision problems		8 (47)	6 (46)	4 (29)	11 (42)	7 (39)
	Dizziness		3 (18)	5 (38)	2 (14)	6 (23)	4 (22)
	**Estimated good health**		8 (47)	8 (62)	5 (36)	17 (65)	9 (50)
		Estimated better health compared with contemporary	8 (47)	3 (23)	5 (36)	11 (42)	5 (28)
	Estimated good balance		5 (29)	3 (23)	4 (29)	7 (27)	5 (28)
	Feel pain daily		4 (24)	2 (15)	3 (21)	5 (19)	4 (22)
**Physical activity questions, n (%)**							
	Practice some physical activity		7 (41)	6 (46)	3 (21)	11 (42)	5 (28)
	Practiced some sport in the past		10 (59)	8 (62)	10 (71)	14 (54)	9 (50)
	Practiced strength exercises in the past		3 (18)	6 (46)	3 (21)	6 (23)	6 (33)

^a^A fall was defined as an event, which resulted in a person coming to rest on the ground or other lower level.

### Program Adherence

The median relative training adherence was 59.3% in the brochure group (IQR 0.0%-88.9%), 84.0% in the social group (IQR 77.2%-89.5%), and 80.9% (IQR 52.8%-88.9%) in the individual group. We registered 7 active (41%) and 10 inactive (59%) participants for the brochure group (n=17), 11 active (85%) and 2 inactive (15%) participants for the social group (n=13), and 8 active (57%) and 6 inactive (43%) participants for the individual group (n=14). In total, 26 of 44 participants reached the goal of 75% adherence or more and were analyzed as active (59%), whereas 18 of 44 (41%) were classified as poor compliers and inactive participants. The brochure group had more inactive participants, but this did not meet statistical significance (*U=*167, *P*=.06, *r=*.29). There were no significant differences between the 2 tablet groups. By further investigating differences in baseline characteristics between active and inactive participants, we found significantly more inactive participants with type II diabetes mellitus (*U=*178, *P*=.03, *r=*.34). There were no further differences between active and inactive participants concerning their baseline characteristics. Details on the 25% attrition rate have been previously published [53].

### Gait Analysis

Table 3 details the results of the spatiotemporal walking parameters of the 3 groups. Participants’ performance in the posttest was significantly higher than in the pretest throughout all 4 conditions (preferred walking, fast walking, preferred dual task walking, fast dual task walking) for the 2 tablet groups (social group and individual group). In contrast, apart from step length during fast walking, there were no significant improvements in the brochure group observable. The active participants performed significantly better at posttests compared to pretests, whereas the inactive participants did not improve.

Differences between the brochure group and the tablet groups, between the 2 tablet groups, and between active and inactive participants are summarized in Table 4. Performance of the tablet groups differed significantly from the brochure group in the dual task walking condition with preferred walking speed: dual task preferred walking (velocity: *U=*138.5, *P*=.03, *r=*.33; cadence: *U=*138.5, *P*=.03, *r=*.34). Preferred, fast walking, and dual task preferred walking did not show significant differences between the 2 tablet groups (social group vs individual group). However, a significant difference was found for dual task fast walking (SD of step length: *U=*49, *P*=.04, *r=*.39). Comparison between active and inactive participants revealed significant differences in velocity throughout all conditions (preferred: *U=*145, *P*=.03, *r=*.32; fast: *U=*146.5, *P*=.04, *r=*0.32; dual task preferred walking: *U=*82.5, *P*>.001, *r=*.55; dual task fast walking: *U=*100.5, *P*=.001, *r=*.05). Although the active participants outperformed the inactive participants in most parameters in walking conditions, preferred walking, dual task preferred walking, and in dual task fast walking, there were no further significant differences for fast walking.

Analyses of dual task costs (DTC) with preferred walking speed revealed significant differences between pretest and posttest for the individual group (velocity: *P*=.03, *z*=–2.134; cadence: *P*=.02, *z*=–2.401; step time: *P*=.02, *z*=–2.401; double support time: *P*=.02, *z*=–2.401). In contrast, performance over time did not increase for the brochure and social groups. In the fast walking condition, DTC decreased for the brochure group (SD of step time: *P*=.047, *z*=1.988). No significant differences were reported between (1) the brochure group and the tablet groups, and (2) the social group and the individual group.

Between-group differences in DTC of walking revealed significant greater performance for the active group when compared with the inactive group (DTC preferred: velocity: *U=*151.5, *P*=.047, *r=*0.30; cadence: *U=*139.5, *P*=.02, *r=*.34; step time: *U=*149.5, *P*=.04, *r=*.31; DTC fast: SD of step time: *U=*152.5, *P*=.049, *r=*.30).

**Table 3:** Participants’ single and dual task walking performance during the pretests and posttests and within-group significance calculated with Wilcoxon signed rank test.

Condition and parameters			Single task walking, median (IQR)			Dual task walking, median (IQR)		
			Pretest	Posttest	*P*	Pretest	Posttest	*P*
**Brochure group**								
	**Preferred**							
		Velocity (cm/s)	109.9 (103.7,112.1)	109.9 (106.8, 125.4)	.09	86.5 (84.3, 103.6)	86.5 (81.3, 106.1)	.29
		Cadence (steps/min)	109.7 (105.3, 110.1)	109.7 (109.7, 114.1)	.07	91.9 (86.9, 105.5)	91.9 (91.9, 108.1)	.45
		Step length (cm)	60.0 (60.0, 65.4)	60.0 (60.0, 67.2)	.06	55.6 (55.6, 60.0)	55.6 (55.6, 64.1)	.11
		Step time (s)	0.55 (0.55, 0.57)	0.55 (0.52, 0,55)	.07	0.72 (0.57, 0.73)	0.72 (0.56, 0.72)	.33
		SD step length (cm)	2.70 (2.38, 2.74)	2.70 (2.03, 2.70)	.24	2.93 (2.32, 2.93)	2.93 (2.49, 3.14)	.88
		SD step time (s)	0.022 (0.018, 0.022)	0.022 (0.013, 0.022)	.51	0.107 (0.030, 0.107)	0.107 (0.020, 0.107)	.14
		Double support time (s)	0.35 (0.32, 0.36)	0.35 (0.32, 0.35)	.14	0.50 (0.36, 0.50)	0.50 (0.35, 0.50)	.45
	**Fast**							
		Velocity (cm/s)	142.3 (139.8, 145.0)	142.3 (142.3 (160.0)	.06	97.0 (87.8, 106.1)	97.0 (97.0, 117.4)	.07
		Cadence (steps/min)	127.8 (121.5, 127.8)	127.8 (125.4, 128.7)	.20	97.4 (93.3, 111.3)	97.4 (97.4, 114.6)	.20
		Step length (cm)	66.7 (66.3, 70.5)	66.6 (66.6, 74.1)	.02	59.3 (59.3, 64.0)	59.3 (59.3, 66.5)	.06
		Step time (s)	0.48 (0.48, 0.49)	0.48 (0.47, 0.48)	.17	0.65 (0.54, 0.66)	0.65 (0.52, 0.65)	.22
		SD step length (cm)	2.96 (2.65, 2.96)	2.96 (2.66, 3.16)	.24	3.44 (2.99, 4.07)	3.44 (2.77, 3.46)	.45
		SD step time (s)	0.020 (0.013, 0.020)	0.020 (0.011, 0.020)	.65	0.072 (0.022, 0.072)	0.072 (0.019, 0.072)	.11
		Double support time (s)	0.27 (0.27, 0.27)	0.27 (0.25, 0.27)	.17	0.42 (0.31, 0.44)	0.42 (0.31, 0.43)	.24
**Social group**								
	**Preferred**							
		Velocity (cm/s)	108.3 (95.87, 129.73)	121.8 (106.4, 143.3)	.004	91.3 (70.4, 111.8)	115.6 (85.1, 130.5)	.02
		Cadence (steps/min)	106.1 (101.1, 112.3)	116.6 (105.7, 117.7)	.006	93.4 (86.9, 105.1)	107.1 (91.6, 117.9)	.01
		Step length (cm)	60.9 (54.6, 67.3)	60.9 (58.9, 75.1)	.004	58.2 (50.6, 64.9)	58.2 (50.2, 69.5)	.11
		Step time (s)	0.57 (0.54, 0.59)	0.51 (0.51, 0.57)	.01	0.65 (0.57, 0.69)	0.56 (0.51, 0.66)	.01
		SD step length (cm)	2.05 (1.80, 2.21)	1.70(1.38, 2.14)	.37	2.38 (1.92, 2.74)	2.40 (1.52, 2.61)	.29
		SD step time (s)	0.019 (0.015, 0.021)	0.015 (0.011, 0.020)	.14	0.033 (0.019, 0.041)	0.023 (0.013, 0.040)	.03
		Double support time (s)	0.33 (0.30, 0.38)	0.32 (0.27, 0.35)	.02	0.38 (0.35, 0.46)	0.33 (0.28, 0.45)	.11
	**Fast**							
		Velocity (cm/s)	146.5 (130.6, 182.8)	152.8(136.3, 200.5)	.03	117.3 (101.9, 135.5)	141.1 (108.9, 158.7)	.003
		Cadence (steps/min)	128.5 (121.2, 136.2)	133.6 (119.8, 154.7)	.06	107.7 (105.7, 118.7)	115.3 (106.5, 132.8)	.003
		Step length (cm)	69.6 (59.7, 76.5)	69.4 (65.4, 78.3)	.42	63.9 (56.1, 70.2)	63.9 (58.8, 72.0)	.09
		Step time (s)	0.47 (0.44, 0.49)	0.50 (0.39, 0.50)	.05	0.56 (0.51, 0.59)	0.52 (0.45, 0.57)	.003
		SD step length (cm)	2.62 (20.5, 3.27)	2.66 (1.86, 3.11)	.59	2.01 (1.76, 2.50)	2.54 (2.28, 3.04)	.05
		SD step time (s)	0.014 (0.011, 0.018)	0.014 (0.010, 0.017)	.89	0.019 (0.014, 0.033)	0.019 (0.015, 0.034)	.56
		Double support time (s)	0.25 (0.20, 0.27)	0.23 (0.17, 0.27)	.25	0.30 (0.28, 0.36)	0.31 (0.24, 0.36)	.01
**Individual group**								
	**Preferred**							
		Velocity (cm/s)	123.0 (112.9, 137.2)	132.8 (123.0, 156.1)	.01	100.8 (91.7, 109.6)	112.3 (95.7, 140.1)	.01
		Cadence (steps/min)	113.7 (109.2, 119.3)	124.4 (113.6, 128.9)	.01	102.6 (91.1, 109.6)	109.2 (90.5, 127.5)	.004
		Step length (cm)	64.8 (61.6, 70.4)	64.8 (64.1, 70.4)	.08	60.2 (55.7, 62.8)	60.9 (60.0, 65.2)	.02
		Step time (s)	0.53 (0.50, 0.55)	0.48 (0.47, 0.53)	.01	0.59 (0.55, 0.71)	0.57 (0.47, 0.66)	.01
		SD step length (cm)	1.88 (1.68, 2.10)	1.74 (1.48, 1.86)	.18	2.79 (2.13, 3.46)	2.79 (1.99, 2.94)	.29
		SD step time (s)	0.017 (0.015, 0.019)	0.013 (0.011, 0.017)	.01	0.032 (0.023, 0.440)	0.021 (0.023, 0.222)	.003
		Double support time (s)	0.30 (0.27, 0.33)	0.27 (0.23, 0.30)	.01	0.37 (0.32, 0.45)	0.32 (0.25, 0.40)	.004
	**Fast**							
		Velocity (cm/s)	179.1 (167.2, 190.9)	183.8 (175.0, 216.1)	.03	134.9 (122.2, 142.6)	140.3 (125.8, 172.0)	.11
		Cadence (steps/min)	146.4 (141.4, 154.6)	155.7 (146.4, 171.1)	.01	123.3 (113.1, 132.0)	125.8 (118.0, 144.1)	.09
		Step length (cm)	73.6 (69.7, 75.4)	73.6 (70.2, 76.6)	.53	68.4 (61.8, 68.7)	67.4 (66.3, 68.7)	.79
		Step time (s)	0.41 (0.39, 0.42)	0.39 (0.35, 0.41)	.01	0.49 (0.45, 0.56)	0.48 (0.42, 0.53)	.11
		SD step length (cm)	2.72 (2.34, 3.04)	2.72 (2.34, 3.97)	.79	2.96 (2.18, 4.02)	2.87 (2.53, 3.02)	.33
		SD step time (s)	0.014 (0.011, 0.017)	0.012 (0.010, 0.014)	.37	0.029 (0.018, 0.051)	0.018 (0.014, 0.036)	.02
		Double support time (s)	0.19 (0.16, 0.20)	0.16 (0.12, 0.19)	.04	0.24 (0.28, 0.29)	0.24 (0.19, 0.27)	.13
**Active**								
	**Preferred**							
		Velocity (cm/s)	117.1 (104.5, 129.3)	128.1 (107.8, 153.5)	>.001	104.3 (68.8, 114.0)	121.0 (93.3, 136.1)	>.001
		Cadence (steps/min)	109.1 (102.1, 118.4)	107.1 (116.7, 126.1)	>.001	102.6 (88.1, 109.0)	111.7 (93.2, 126.3)	>.001
		Step length (cm)	64.6 (58.4, 69.3)	65.8 (60.8, 74.4)	>.001	59.9 (52.2, 66.7)	64.1 (56.3, 69.5)	.001
		Step time (s)	0.55 (0.51, 0.59)	0.51 (0.48, 0.56)	>.001	0.58 (0.55, 0.68)	0.54 (0.48, 0.65)	>.001
		SD step length (cm)	2.06 (1.82, 2.41)	1.71 (1.40, 2.17)	.03	2.48 (2.00, 3.21)	2.48 (1.64, 2.94)	.05
		SD step time (s)	0.017 (0.014, 0.021)	0.012 (0.011, 0.018)	.01	0.030 (0.019, 0.040)	0.018 (0.013, 0.037)	>.001
		Double support time (s)	0.31 (0.29, 0.37)	0.29 (0.25, 0.38)	.001	0.27 (0.32, 0.48)	0.32 (0.27, 0.42)	.001
	**Fast**							
		Velocity (cm/s)	157.3 (141.0, 181.8)	166.9 (145.7, 204.9)	.001	122.8 (99.8, 10.3)	142.6 (106.9, 172.0)	>.001
		Cadence (steps/min)	131.1 (119.8, 145.9)	135.7 (121.7, 163.5)	.002	111.9 (102.1, 124.0)	121.8 (107.2, 139.1)	>.001
		Step length (cm)	71.8 (63.0, 77.6)	72.7 (67.0, 81.6)	.03	64.4 (58.9, 69.8)	66.8 (60.3, 74.4)	.02
		Step time (s)	0.46 (0.41, 0.50)	0.44 (0.37, 0.49)	.002	0.54 (0.48, 0.59)	0.49 (0.43, 0.57)	>.001
		SD step length (cm)	2.66 (2.13, 3.49)	2.62 (1.93, 3.33)	.88	2.63 (1.80, 4.11)	2.68 (2.12, 3.40)	.68
		SD step time (s)	0.013 (0.011, 0.016)	0.011 (0.009, 0.018)	.96	0.019 (0.016, 0.038)	0.017 (0.013, 0.025)	.002
		Double support time (s)	0.24 (0.19, 0.27)	0.22 (0.13, 0.26)	.02	0.29 (0.26, 0.37)	0.28 (0.20, 0.36)	.001
**Inactive**								
	**Preferred**							
		Velocity (cm/s)	109.9 (104.9, 116.7)	109.9 (109.9, 125.4)	.09	86.5 (85.4, 100.1)	86.5 (85.8, 93.5)	.87
		Cadence (steps/min)	109.7 (106.9, 113.7)	111.3 (109.7, 114.1)	.06	91.9 (89.9, 98.4)	91.9 (89.1, 98.4)	.75
		Step length (cm)	60.0 (60.0, 64.1)	60.4 (60.0, 64.8)	.09	55.6 (55.6, 60.2)	55.6 (55.6, 60.6)	.74
		Step time (s)	0.55 (0.53, 0.56)	0.54 (0.53, 0.55)	.04	0.72 (0.65, 0.72)	0.72 (0.65, 0.72)	.74
		SD step length (cm)	2.56 (1.91, 2.70)	2.39 (1.82, 2.70)	.99	2.79 (2.37, 2.93)	2.93 (2.72, 2.95)	.18
		SD step time (s)	0.022 (0.017, 0.022)	0.019 (0.017, 0.022)	.31	0.107 (0.046, 0.184)	0.107 (0.039, 0.184)	.40
		Double support time (s)	0.35 (0.32, 0.35)	0.34 (0.30, 0.35)	.18	0.48 (0.40, 0.50)	0.50 (0.40, 0.50)	.87
	**Fast**							
		Velocity (cm/s)	142.2 (142.2, 179.1)	144.4 (142.2, 179.1)	.18	97.0 (97.0, 125.3)	98.4 (97.0, 134.9)	.61
		Cadence (steps/min)	127.8 (127.4, 144.5)	128.2 (127.8, 146.4)	.09	97.4 (97.4, 119.4)	101.7 (97.4, 119.4)	.74
		Step length (cm)	66.6 (66.6, 73.6)	67.0 (66.6, 73.6)	.61	59.6 (59.3, 68.3)	60.5 (59.3, 67.7)	.99
		Step time (s)	0.48 (0.42, 0.48)	0.47 (0.41, 0.48)	.09	0.65 (0.53, 0.65)	0.62 (0.53, 0.65)	.87
		SD step length (cm)	2.93 (2.70, 2.96)	2.96 (2.72, 2.96)	.31	3.24 (2.61, 3.44)	3.25 (2.78, 3.44)	.87
		SD step time (s)	0.017 (0.014, 0.020)	0.018 (0.014, 0.020)	.50	0.071 (0.033, 0.072)	0.060 (0.032, 0.072)	.74
		Double support time (s)	0.027 (0.019, 0.027)	0.026 (0.019, 0.027)	.18	0.42 (0.29, 0.42)	0.39 (0.25, 0.42)	.99

**Table 4:** *P* values of participants’ walking performance (between-groups differences after intervention phase calculated with Mann-Whitney *U* test).

Condition/parameters		Brochure vs social and individual		Social vs individual		Active vs inactive	
		*P*	*r* ^a^	*P*	*r* ^a^	*P*	*r* ^a^
**Preferred**							
	Velocity	.08	.26	.96	.01	.03	.32
	Cadence	.09	.26	.63	.09	.06	.29
	Step length	.22	.18	.53	.12	.02	.36
	Step time	.14	.22	.92	.02	.06	.28
	SD step length	.91	.02	.99	.00	.03	.33
	SD step time	.08	.26	.44	.15	.04	.30
	Double support time	.19	.20	.53	.12	.01	.38
**Fast**							
	Velocity	.37	.14	.96	.01	.04	.32
	Cadence	.09	.25	.66	.08	.08	.27
	Step length	.38	.13	.96	.01	.07	.27
	Step time	.24	.18	.90	.02	.10	.25
	SD step length	.53	.10	.96	.01	.66	.07
	SD step time	.40	.13	.44	.15	.58	.08
	Double support time	.36	.14	.72	.07	.12	.23
**Dual task preferred**							
	Velocity	.03	.33	.44	.15	<.001	.55
	Cadence	.03	.33	.37	.17	<.001	.57
	Step length	.37	.14	.99	.00	.003	.45
	Step time	.05	.29	.47	.14	.001	.48
	SD step length	.28	.16	.92	.02	.01	.40
	SD step time	.20	.19	.47	.14	.002	.47
	Double support time	.11	.24	.17	.26	.003	.47
**Dual task fast**							
	Velocity	.20	.19	.59	.10	.001	.49
	Cadence	.07	.28	.59	.10	.001	.50
	Step length	.58	.08	.20	.25	.10	.25
	Step time	.14	.22	.47	.14	.001	.48
	SD step length	.30	.16	.04	.39	.76	.05
	SD step time	.61	.08	.38	.17	.01	.42
	Double support time	.22	.18	.96	.01	.002	.47

^a^Effect size (small effect: *r*=.1; medium effect: *r*=.3; large effect: *r*=.5).

### Physical Performance and Fear of Falling

Table 5 demonstrates changes over time for fear of falling (FES-I) and physical performance (SPPB) for the 3 groups. The SPPB showed significant improvements for all groups. There were no differences between pretest and posttest for FES-I. Significant group differences for FES-I were observed between the brochure group and tablet groups (*U=*151.5, *P*=.04, *r=*.31); however, not between the 2 tablet groups (*U=*89.5, *P*=.94, *r=*.01) or the active and inactive participants (*U=*210.5, *P*=.53, *r=*.09). We found a significant difference between active and inactive participants in SPPB (*U=*139, *P*=.02, *r=*.36).

**Table 5:** Physical performance and fear of falling during the pretest and the posttest and significance of within-group differences pre-post calculated with Wilcoxon signed rank test.

Test	Brochure group, median (IQR)			Social group, median (IQR)			Individual group, median (IQR)		
	Pretest	Posttest	*P*	Pretest	Posttest	*P*	Pretest	Posttest	*P*
SPPB	9.8 (9.4, 11.0)	11.0 (9.8, 12.0)	.02	9.7 (8.0, 11.0)	12.0 (9.7, 12.0)	.02	9.9 (8.8, 11.0)	11.0 (9.9, 12.0)	.02
FES-I	17.9 (16.0, 17.9)	17.9 (17.0, 17.9)	.49	20.0 (17.0, 21.5)	20.0 (17.0, 20.6)	.23	18.9(17.5, 20.0)	18.0 (16.0, 18.9)	.27

### Social Interaction

We registered the number of dispatched messages. The total number of messages dispatched to the bulletin board was 31 from the social group participants sent by 8 of 13 social group participants. The caregivers dispatched a total of 37 messages to the bulletin board. Six of 13 social group participants wrote 13 messages to another participant. Participants received 84 messages from caregivers; 93 messages were dispatched by 11 social group participants to caregivers. Thus, most interaction occurred between caregivers and participants and not between participants, indicating the importance of social support from caregivers.

## Discussion

### Principal Findings

This study compared 3 different home-based training programs and their effect on measures of gait quality while considering adherence to the training program. We hypothesized that there would be differing results for (1) the tablet-based groups when compared to the brochure group, (2) the tablet group with social motivation strategies when compared to the tablet group with individual motivation strategies, and (3) active participants when compared to inactive participants. The outcomes of interest were gait quality and lower extremity physical performance. Furthermore, the aim was to assess the influence of different motivation strategies offered to the trainees.

### Gait Analysis

From previous studies, we know that home-based exercise training can have beneficial effects on physical performance outcomes [1,75], provided the program is adhered to [76]. Our results of the walking quality analysis show significant improvements from pretest to posttest, especially in the training groups that showed high adherence rates. The tablet groups reached higher adherence rates compared to the brochure group. Furthermore, participants in the tablet groups were able to improve gait velocity throughout all walking conditions (preferred and fast single task walking, preferred and fast dual task walking), whereas the brochure group failed to increase this performance aspect following 12 weeks of training. Usual gait speed is a predictor for disability, falls, and mortality [26]. In comparison to our brochure group and the inactive participants, the tablet groups and the active participants reached improvements of 10 cm/s or more. Such improvements represent clinically meaningful change in gait speed [26]. Walking at fastest speed may serve as a useful diagnostic measure for people at higher risk for multiple falls. In the fast walking condition, shorter step length relates to falls [77]. We reported an improvement in step length during walking in our group of active participants, but not in the inactive participants. Both the tablet groups and the active participants improved velocity during fastest walking. Compared to literature reference values where an expected preferred and fast walking speed for independently living elderly would be approximately 133 cm/s and 207 cm/s, respectively [78], our samples performed worse pretraining. Following training, however, the tablet groups improved toward these reference values.

Frail elderly people and elderly people who tend to fall exhibit increased variability in measures of gait [23,79,80]. Elderly nonfallers present low rates of variability of temporal variables [20,24]. Decreased leg strength explains greater variability [81]. This study shows that tablet-based exercise may decrease gait variability provided the trainees adhere to the training plan. The brochure group demonstrated no decrease in gait variability after the intervention. In contrast, the tablet groups showed significantly lower variability throughout all measurement conditions. This especially holds true for the group with individual motivation strategies and for step time variability. Step time and double support time—factors that have been previously related to falls [35]—decreased throughout all conditions, again solely in the tablet groups. Thus, our trial underpins the importance of training program compliance in preventive exercise programs for elderly and indicates that an appropriate targeted tablet-based exercise application is able to positively influence exercise adherence in independent-living elderly training at home. Because of the higher training adherence, the tablet-based exercise groups improved their single and dual task walking to a larger extent compared to a group trained with a more conventional type of brochures-based training.

Dual task walking (ie, the ability to perform a second task while walking) is a key element to remain independent because this is an ability required for many activities in daily life. Daily activities pose high cognitive demands and safe walking should be practicable under cognitively distractive or otherwise challenging conditions. Our findings in dual task walking are similar to some extent to the findings of Pichierri et al [82], who reported no improvements in dual task walking with an isolated motor training program. This finding was in-line with previous studies that investigated the effect of an isolated physical training program that were not able to demonstrate improvements in walking under attention-demanding circumstances [83,84]. Our intervention did not consist of a cognitive training part and it can be speculated that an extension of our program with a cognitive challenge will be more effective in influencing walking under attention-demanding circumstances. Future research should be directed to investigating the value of additional cognitive elements to the training program to substantiate these assumptions.

### Physical Performance

We found a significant improvement in SPPB scores within all groups, reflecting enhanced lower extremity function and walking ability [85]. On average, a person that reaches less than 10 points on the SPPB is almost 3.5 times more susceptible to suffer from mobility disability than a person scoring the maximum of 12 points [85]. All 3 groups reached a median relative score of 11 points or more in the posttests, compared to a median relative score of less than 10 points in the pretests.

### Program Adherence

An important issue in the field of exercise interventions with elderly people is adherence to the training plan [76]. Elderly people will only be able to reap the gains from exercise under the precondition that they comply with and progress through the exercise plan. A systematic review investigating adherence to multifactorial interventions in falls prevention in community settings for clinical trials reported rates ranging between 28% and 95%. The general range was approximately 75%, which was the reason we chose this level to divide our training group into active versus inactive participants. Compared with these values [86], we achieved better or similar rates as 75% adherence; however, this was for the tablet-based training groups only. Furthermore, we observed the most prominent differences in training effects between the active and the inactive participants. Active participants demonstrated significantly higher performance in several spatial-temporal walking parameters compared to the inactive participants. This supports findings from other studies showing that better compliance leads to significantly higher training-related benefits [87,88] and indicates that adherence moderated treatment effectiveness. We report on values after 3 training months, but Nyman and Victor [64] reported values that may be expected by 12 months. In a future phase III trial, the follow-up period for the assessment of adherence and attrition should preferably be extended to a similar time frame to facilitate comparability of this future study with reference values.

Social support [48] and commitment to or advice from health experts, physicians, or caregivers are reasons for higher compliance rates and more moderate exercise conduction [44,89]. In an analysis of compliance in home-based exercise programs, an increase in compliance was registered in a brochure-based group compared with the outcome of a control group who did not receive any recommendations [90]. Moreover, a DVD-supported training program reported better adherence compared to brochures [89]. DVDs might help to overcome motivational problems [89] and enhance exercise correctness [91] compared to brochure-guided exercise programs. The amount of messages dispatched indicates that most interactions occurred between caregivers and participants and not between participants. This reflects the importance of social support of caregivers to the trainees.

Motivation is an important parameter for home-based exercise performance [92] and should be explicitly considered in the design of interventions. The program used in our study explicitly considered motivational elements and allowed participants contacting experts and training partners. The most active participants were found in the social group, whereas the most inactive participants belonged to the brochure group (although this did not meet statistical significance). This result supports our assumption that social motivation strategies enhance compliance. Apart from that, there seems to be no direct gain from social motivation strategies on walking quality compared with individual motivation strategies because the results of the 2 tablet groups did not differ in the outcome measures.

### Limitations

An obvious limitation of this study is that the groups were only partly randomized. Therefore, this study only reveals first estimates and warrants further research with a properly randomized model. A further limitation is the rather small sample size. Measurements of compliance are based on written information of participants, which cannot be seen as an instrument that guarantees the participants followed the exercises. Better control instruments would be a useful extension to further studies.

Additionally, correctness of the exercise was not controlled. To overcome this problem, further research should include technologies to control posture and movement pattern. Video analysis with 3-dimensional motion tracking equipment or microelectromechanical systems (MEMS) can offer opportunities to link clinicians and potential users [93]. Another option is the Health Hub (HH) software that allows recognition and analysis of motion [93].

We treated the dropouts of this study as part of the treatment group to which they were assigned even if they did not receive the full intervention. Intention-to-treat is a recommended approach to several types of nonadherence to the study protocol [94], able to reduce the potential dropout bias effect [95]. We replaced missing data with the mean values of the groups, thus allowing complete case analysis. A drawback of this approach is reduced variability and weakening of covariance and correlation estimates in the data. Future adequately powered studies with larger samples should be performed with both intention-to-treat and per-protocol analysis.

### Conclusions

The findings of this study are in-line with previous research that demonstrated improvements in gait quality and physical performance of older adults after strength-balance exercises. This study adds useful information about home-based training programs for older adults. Our participants adhered better to the weekly physical intervention when provided with the ActiveLifestyle app. This clearly described exercise program, including motivational aspects, an attractive design, automatized reminders, and the opportunity to give feedback about performed exercises to training supervisors, seems to contain important elements to enhance adherence and compliance rates, which leads to training-related improvements. The trainees that complied with the training plan improved gait and physical performance. The tablet-based program resulted in higher rates of adherence compared to the brochure-based program. These findings suggest that in older adults a tablet-based intervention may enhance compliance and potentially offers an effective way to improve gait.

## Abbreviations

DTC: dual task costs
FES-I: Falls Efficacy Scale International
HH: Health Hub
MEMS: microelectromechanical systems
SPPB: Short Physical Performance Battery
